# Nighttime Sleep Awakening Frequency and Its Consistency Predict Future Academic Performance in College Students

**DOI:** 10.3390/ijerph19052933

**Published:** 2022-03-02

**Authors:** Ghee Wee Ho, Zhenzhi Yang, Linna Xing, Ken Kang-Too Tsang, Huada Daniel Ruan, Yu Li

**Affiliations:** 1 Applied Psychology Programme, Division of Science and Technology, BNU-HKBU United International College, 2000 Jintong Rd, Tangjiawan, Zhuhai 519087, China; jennyyang@uic.edu.cn (Z.Y.); solnalnxing@uic.edu.cn (L.X.); 2 Statistics Programme, Division of Science and Technology, BNU-HKBU United International College, Zhuhai 519087, China; k2zeng@icloud.com; 3 Environmental Science Programme, Division of Science and Technology, BNU-HKBU United International College, Zhuhai 519087, China; hruan@uic.edu.cn

**Keywords:** sleep, academic performance, grade point average, college students, wearable device, longitudinal, nighttime sleep awakening

## Abstract

Although the relationship between sleep and academic performance has been extensively examined, how sleep predicts future academic performance (e.g., 2–3 years) remains to be further investigated. Using wearable smartwatches and a self-report questionnaire, we tracked sleep activities of 45 college students over a period of approximately half a month to see whether their sleep activities predicted their academic performance, which was estimated by grade point average (GPA). Results showed that both nighttime sleep awakening frequency and its consistency in the tracking period were not significantly correlated with the GPA for the courses taken in the sleep tracking semester (current GPA). However, both nighttime sleep awakening frequency and its consistency inversely predicted the GPA for the rest of the courses taken after that semester (future GPA). Moreover, students with more difficulty staying awake throughout the day obtained lower current and future GPAs, and students with higher inconsistency of sleep quality obtained lower future GPA. Together, these findings highlight the importance of nighttime sleep awakening frequency and consistency in predicting future academic performance, and emphasize the necessity of assessing the consistency of sleep measures in future studies.

## 1. Introduction

As a necessity for humans, healthy sleep plays an essential role in maintaining normal brain and cognitive functions [1,2,3,4,5]. For example, it has been revealed that sleep facilitates language learning [6], and consolidates memory [7,8]. Extant studies have provided mounting evidence that individuals who are suffering from sleep disturbances or deprivation exhibit not only severe impairments on various cognitive functions [9,10,11,12,13,14,15,16], but also aberrances in neural structure and activity [17,18,19,20,21,22,23]. In the context of school education, sleep problems or disturbances are prevalent in students of all ages, ranging from primary school [24,25,26] to college [27,28,29,30]. The negative influences of sleep problems on academic performance have also been reported in both children and college students, even though the mechanisms underlying the relationships remain to be explored. It has been widely observed that various sleep-related measures, such as sleep duration and sleep quality, are associated with academic performance [23,31,32,33], indicating the importance of normal sleep patterns in school education.

For college students, grade point average (GPA) is one of the most important indicators reflecting the success of college education. It is associated with various crucial aspects, including intelligence, socio-economic status, schooling quality, learning motivation, and health problems [34,35]. In the past two decades, much research has been conducted to examine the associations between sleep and GPA [33,36,37]. There is increasing evidence that GPA can, to some degree, be predicted by certain sleep measures, including sleep duration [38,39,40,41,42,43,44,45] and sleep quality [42,43,46,47,48,49,50,51].

In the extant literature, most studies investigating the relationships between sleep and academic performance used averages of sleep measures in a period as indexes in data analysis and interpretation [41,42,48,49,52,53,54]. Beyond these traditional sleep measures, sleep consistency or variability have been receiving more and more attention in recent years [41,43,44,53,55,56,57,58]. The consistency of a sleep measure is usually estimated by standard deviation or standard error of the mean in a period (e.g., a few weeks), where a higher value denotes lower consistency or high variability. In the studies of college students, Taylor et al. [41] found that sleep duration consistency estimated by standard deviation showed a significantly negative relationship with GPA. Smarr [58] found that sleep duration consistency estimated by standard error of the mean was negatively associated with class performance. Okano et al. [43] found that sleep duration consistency estimated by standard deviation was negatively associated with GPA. Sridhar et al. [44] found that sleep consistency indexed by standard deviation was negatively correlated with homework grades. Using a sleep regularity index, Phillips et al. [53] found that standard deviations of sleep onset times and wake-up times were negatively correlated with GPA. Collectively, lower sleep consistency and higher variability are associated with lower academic performance, demonstrating that sleep consistency can be a reliable predictor of academic performance.

It is worth noting that these studies have primarily focused on how sleep predicts GPA for the courses taken by students just after sleep tracking. There are only a few studies that used a longitudinal design to examine the relationships between sleep measures and long-term academic performance, such as GPA two years later [59,60,61,62,63]. Of these studies, three studies reported significant associations between sleep and long-term academic performance. Wong et al. [59] found that sleep duration of the first semester predicted the overall GPA across three semesters in college students. Galambos et al. [60] found that overall GPA for four years courses was lower when bedtimes were later in college students. Stormark et al. [63] found that persistent sleep problems across several years elevated the risk of academic performance in children. These findings suggest that sleep activities can serve as good indicators of future or long-term academic performance.

Among the studies examining associations between sleep and academic performance, most used self-report questionnaires, such as the Pittsburgh Sleep Quality Index [64] and in-house ones, to estimate sleep activities. Results from self-reports are inherently subjective, and participants’ responses can be influenced by individual differences in reporting standards, and concurrent emotional states. The field calls for objective approaches. Polysomnography is thought to provide an objective way for tracking sleep activities, and has been widely used in previous research [65,66,67,68,69]. However, its use in laboratories, to a certain degree, has an impact on ecological validity. In recent years, wearable smart devices have been invented and widely used to track physiological activities, such as heart rate, calorie consumption, walking steps, and sleep patterns (e.g., duration, onset time, and offset time). Wearable smart devices can be used at home and school dormitories where participants feel familiar and comfortable, demonstrating high ecological validity. Sleep measures provided by smart devices are estimated based on implemented computer algorithms; individuals’ sleep characteristics can therefore be derived with the very same standards. Because of this, sleep measures generated from the devices could be more objective compared to self-report questionnaires. A few studies have already employed wearable devices to track sleep activity [43,44,50,70,71,72,73]. Out of these studies, four examined the relationships between wearable-device-based sleep measures (e.g., duration and quality) and academic performance. Okano et al. [43] found that better sleep quality, longer sleep duration, and greater sleep consistency were associated with better grades. Sridhar et al. [44] found a non-significant association between average sleep duration and average grades on weekly homework assignments, but a significantly negative association between sleep variability and average homework grades. Wang et al. [73] found that wearable devices with feedback on sleep quality improved both sleep and academic outcomes in students. Adelantado-Renau et al. [50] used both wearable devices and self-reported measures, and found that sleep duration estimated by devices was associated with verbal ability, and self-reported sleep quality was associated with GPA. These provide strong evidence of the usability of wearable devices in predicting academic performance.

In this longitudinal study, we aimed to examine the association between sleep measures and GPA in college students. We used wearable smartwatches to track students’ sleep activities in a short period, and a self-report questionnaire to record students’ subjective feelings about their sleep and daily performance, and collected both their short-term and long-term GPAs. We considered both averages of sleep measures in the sleep tracking period, and their consistency estimated by standard deviations [43], as these two different types of measures could provide a bigger picture of sleep activity. Considering the exploratory nature of this study, we generally hypothesized that sleep measures and their consistency would predict students’ academic performance estimated by GPA. Correlation and mediation analyses were used to determine the relationships between sleep and GPA. The design of the present study is innovative in that it used objective and subjective sleep measures and their consistency, and also considered short-term and long-term academic performance.

## 2. Methods and Materials

### 2.1. Participants

Two separate groups of students volunteered to participate in this study. Group 1 consisted of 25 students (age range, 19.09–20.75 years; mean age, 20.11 years; 17 females), whereas the second group consisted of 22 students (age range, 18.67–21.15 years; mean age, 19.93; 17 females). All the participants were undergraduate students majoring in Applied Psychology in BNU-HKBU United International College. None of them reported any neurological impairments in the past, and all of them had normal hearing and normal or corrected-to-normal vision. The participants were notified that if there were any stressful life events (e.g., important examinations or death of family members) or any somatic or mental diseases (e.g., gastric ulcer, body injury, and clinical depression and anxiety) in the sleep tracking period, they were asked to report to the investigators by filling in a self-report questionnaire (see below). None of them reported any on these. Two students failed to complete the study, and their data were excluded from data analyses. The Human Research Ethics Committee of BNU-HKBU United International College approved this study. A consent form was obtained from participants prior to the study.

### 2.2. Procedure

The two groups of students were being tracked separately. Each of the participants was asked to wear a smartwatch, the “Huawei Honor Watch S1” issued in 2016, to track their daily heart rate (mean, minimum, and maximum), sleep score (calculated with algorithms implemented), sleep time, deep/light/REM sleep time (min), and nighttime sleep awakening frequency. Algorithms and sensors implemented in the smartwatch were used to track individuals’ physiological activities (see the details at https://www.smartwatchspecifications.com/devices/huawei-honor-watch-s1-glory-watch-s1/ accessed on 20 December 2021). This device was widely used to track their heart activities (e.g., rate) and sport activities (e.g., walking steps [74,75]), and has previously demonstrated excellent reliability and sensitivity [74,75,76]. It was therefore chosen to be the instrument for monitoring sleep activities. Both groups were being tracked for a period of approximately two weeks, based on the restrictions on the students’ academic schedules. Both groups were tracked in the middle of the first semester of their second-year study. Group 1 participants were tracked for 13 days, whereas Group 2 participants were tracked for 17 days. On each day of the tracking period, a self-report questionnaire was administered to the participants to collect additional information, including self-reported sleep quality (0–10; 0 denotes worst, and 10 excellent), feeling of tiredness (0–10; 0 denotes worst, and 10 excellent), and whether the participants struggled to keep awake during daytime (0 Yes, 1 No).

After tracking the participants’ sleep, and collecting their answers of the self-report questionnaire, their GPAs for compulsory psychology courses were collected. For each group, we only included compulsory psychology courses to make sure that their GPAs were given based on the same marking criteria. We calculated two types of GPAs, the GPA for the semester courses (current GPA, i.e., the compulsory courses taken in the semester when students were participating in the study: *Biological Psychology*, *Research Methods in Psychology*, and *Developmental Psychology* for Group 1 students; and *Introduction to Psychology* for Group 2 students; ~2 months temporal distance between the sleep tracking period and the final examination of the courses), and the GPA for the courses after that semester (future GPA, i.e., all the compulsory psychology courses taken after the semester: 10 courses for Group 1 and 13 courses for Group 2; ~2.5 years temporal distance between the sleep tracking period and graduation). For the calculation of GPA, marks on final examination, mid-term quiz, and attendance were considered. The GPA for final year project (each student was required to complete a research thesis) were excluded due to its highly subjective rating system. The current GPA was used to determine whether sleep measures could be an indicator of the present academic achievement of students, whereas the future GPA was used to determine whether sleep measures could predict their long-term academic achievement.

### 2.3. Data Analysis

We adopted two approaches for the analyses. First, for each of the sleep measures and self-reported indexes, the data were averaged across the tracking period to generate an overall estimate. We then correlated average values for each of the sleep measures and self-reported indexes to the current and future GPAs. We found that both nighttime sleep awakening frequency and self-reported wakefulness were significantly correlated with the current and future GPAs. Furthermore, nighttime sleep awakening frequency and self-reported wakefulness were also correlated (see Results). To examine these relationships, we further conducted a mediation analysis, specifically by employing the PROCESS macro ([77]) for IBM SPSS Statistics 25.0, to determine whether self-reported wakefulness mediated the influences of sleep awakening frequency on GPA. Second, for each of the sleep measures and self-reported indexes, we calculated the standard deviation of the data in the sleep tracking period to generate an index of consistency (see [43] for a similar approach). Note that for each measure, a large standard deviation denotes low consistency.

## 3. Results

**Figure 1:**
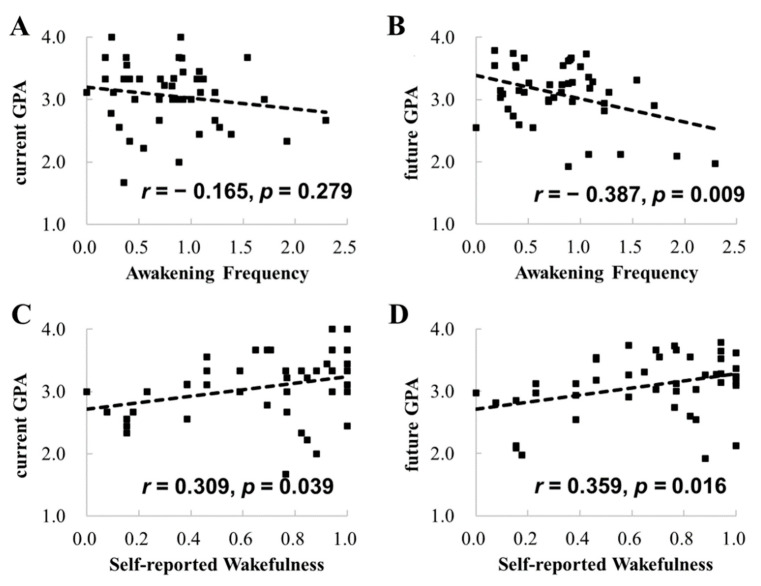
Correlations between awakening frequency and the current and future GPAs (**A**,**B**), and correlations between self-reported wakefulness and the current and future GPAs (**C**,**D**).

The mean of the current GPA was 3.06 (range, 1.67–4.00; *SD*, 0.51), and the mean of the future GPA was 3.08 (range, 1.89–3.77; SD, 0.50). The correlation between the two types of GPAs was statistically significant (*r* = 0.758, *p* < 0.001), indicating a high stability of academic achievement. The statistical details of sleep measures and self-reported measures are presented in Table 1. We first conducted correlational analyses based on the averaged data across the tracking period (see the correlations below the diagonal line in Table 2). The results showed that nighttime sleep awakening frequency was negatively correlated with the future GPA (*r* = −0.387, *p* = 0.009), but not with the current GPA (*r* = −0.165, *p* = 0.279; Figure 1). The correlation between nighttime sleep awakening frequency and the future GPA was still significant after controlling for the current GPA (*r* = −0.408, *p* = 0.006). Hierarchical regression analysis confirmed this by revealing that nighttime sleep awakening frequency significantly accounted for the variance of the future GPA (7.1%, *p* = 0.006) beyond the influences of the current GPA (57.5%; Table 3).

**Figure 2:**
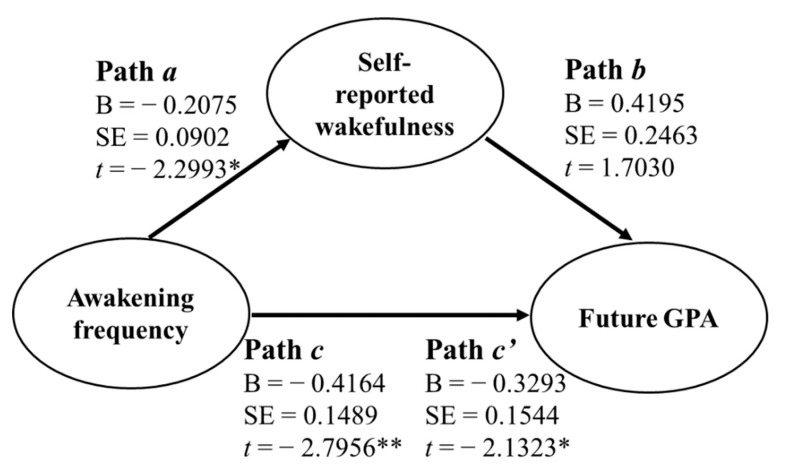
Results of mediation analysis. Path *a* denotes the effect of awakening frequency on self-reported wakefulness. B, regression coefficient. SE, standard error. Path *b* denotes the impact of self-reported wakefulness on the future GPA. Paths *a* and *b* denote the mediated or indirect effect of awakening frequency on the future GPA via self-reported wakefulness. Path *c* denotes the total effects (mediated and direct effects) of awakening frequency on the future GPA. Path *c’* denotes the direct effect of awakening frequency on the future GPA. * *p* < 0.05. ** *p* < 0.01.

For the correlations between GPA and self-reported measures, we found that self-reported wakefulness was positively correlated with both the current GPA (*r* = 0.309, *p* = 0.039) and the future GPA (*r* = 0.359, *p* = 0.016; Figure 1). Moreover, we found that nighttime sleep awakening frequency was negatively correlated with self-reported wakefulness (*r* = −0.331, *p* = 0.026). The findings indicate a possibility that self-reported wakefulness mediated the influences of nighttime sleep awakening frequency on the future GPA. To examine the possibility, we conducted a mediation analysis. As displayed in Figure 2, Path *c* denotes the total effects (mediated and direct effects) of awakening frequency on the future GPA, and Path *c’* denotes the direct effect of nighttime sleep awakening frequency on the future GPA. The mediated effect, i.e., the effect of awakening frequency on the future GPA via self-reported wakefulness, is determined by the difference between Path *c* and Path *c’* or the product of Path *a* and Path *b*. The mediation analysis revealed that Path *c’* was statistically significant (Figure 2), but the difference between Path *c* and Path *c’* was statistically insignificant (Sobel test, *z* = −1.369, *p* = 0.171). These findings suggests that self-reported wakefulness did not significantly mediate the influences of nighttime sleep awakening frequency on the future GPA.

**Figure 3:**
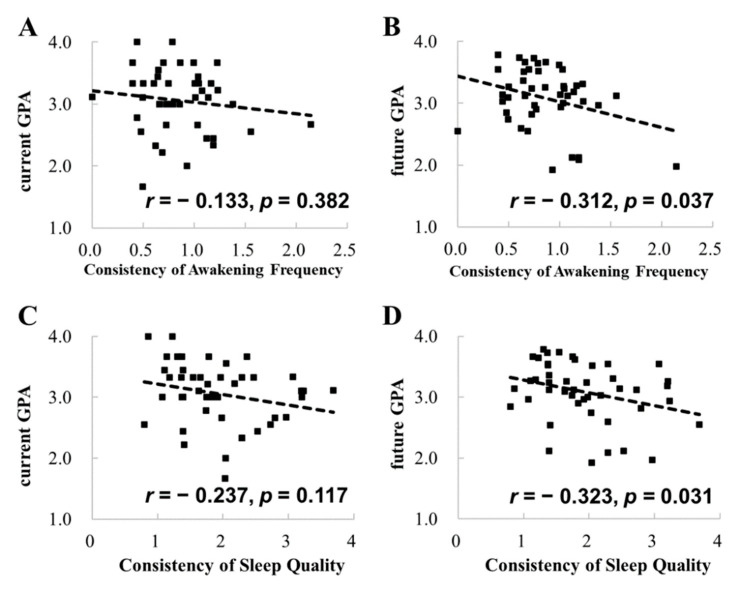
Correlations between the consistency of awakening frequency and the current and future GPAs (**A**,**B**), and correlations between the consistency of self-reported sleep quality and the current and future GPAs (**C**,**D**).

We then conducted correlational analyses based on the consistency of each sleep measure estimated by the standard deviation of the data in the tracking period (see the correlations above the diagonal line in Table 2). The results showed that the consistency of nighttime sleep awakening frequency was positively correlated with the future GPA (*r* = −0.312, *p* = 0.037; Figure 3), but not with the current GPA (*r* = −0.133, *p* = 0.382), indicating that the higher the consistency of nighttime sleep awakening frequency, the higher the future GPA. The correlation between the consistency of nighttime sleep awakening frequency and the future GPA was still significant after controlling for the current GPA (*r* = −0.326, *p* = 0.031). Hierarchical regression analysis confirmed this by revealing that that the consistency of nighttime sleep awakening frequency significantly accounted for the variance of the future GPA (4.5%, *p* = 0.031) beyond the influences of the current GPA (Table 3).

Furthermore, we found that self-reported sleep quality was negatively correlated with the future GPA (*r* = −0.323, *p* = 0.031; Figure 3), but not with the current GPA (*r* = −0.237, *p* = 0.117), indicating that inconsistent sleep time in a period is not associated with lower immediate academic achievement, but long-term academic achievement.

## 4. Discussion

In the present longitudinal study, we used wearable smart wrist watches to examine whether sleep is associated with GPA in college students. We found that nighttime sleep awakening frequency and consistency were negatively correlated with the future GPA, that self-reported daytime wakefulness was correlated with both semester and future GPAs, and that self-reported sleep quality consistency was negatively correlated with the future GPA. These results and possible implications are discussed below.

We found that students with more frequent nighttime sleep awakenings tended to get lower GPAs for future college courses. To the best of our knowledge, this relationship was reported for the first time. Previous studies have tried to measure nighttime sleep awakenings in children [78,79,80,81,82] and college students [83], but these did not reveal significant associations with academic performance, except for El-Sheikh et al. [79]. In the study, El-Sheikh et al. [79] monitored sleep disruptions by using actigraphs in primary school children, and found that sleep quality and duration, which was significantly contributed to by duration after sleep onset, were associated with children’s academic functioning reported by their teachers, despite a direct correlation not being observed between duration after sleep onset and children’s academic functioning. Velten-Schurian et al. [80] found that in children diagnosed with insomnia, those with more parent-reported nighttime awakenings tend to exhibit more daytime sleepiness. It has been previously reported that college students’ daytime sleepiness is associated with GPA [40,84] and exam struggles [85]. The current study revealed that college students who have more difficulties staying awake throughout the day tended to get lower current and future GPAs, indicating the predictive power of daytime sleepiness in concurrent and future academic performance.

Considering that self-reported daytime wakefulness was significantly associated with the future GPA, we further conducted a mediation analysis to determine whether daytime wakefulness mediated the influences of awakening frequency on the future GPA. The results showed that the mediated effect was not significant, indicating that daytime wakefulness and nighttime sleep awakening frequency are not fully overlapped with each other in contributing to academic performance. Other factors, such as daily schedule, emotional arousal, and caffeine intake, may play an important role in between, which remains to be investigated.

The future GPA was significantly correlated not only with nighttime sleep awakening frequency, but also with its consistency. Sleep consistency or variability has been found to be associated with academic performance for sleep duration [41,43,44,58] and sleep onset and wake-up times [53]. Extending these studies, the current study provided a new indicator of academic performance. More importantly, unlike these studies in which a significant relationship between sleep consistency and concurrent GPA was revealed, we determined that the consistency of nighttime sleep awakening frequency predicted future GPA. It suggests that the consistency of nighttime sleep awakening frequency could serve as a longitudinal indicator of academic performance. There is a possible reason why we did not find correlations between these awakening frequency measures and the current GPA: the number of the courses for calculating the current GPA was much less than that of the courses for calculating the future GPA, and thus led to an unreliable estimate for academic performance.

It is worth noting that, similar to the current study, Okano et al. [43] and Sridhar et al. [44] also used wearable activity trackers to monitor sleep activity. Previous studies have demonstrated that electronic devices can accurately track sleep activity in students [50,70,71,72,73]. For example, Beattie et al. [71] demonstrated that algorithms used for Fitbit wearable devices accurately estimated sleep duration and quality in adults with normal sleep. These endeavors demonstrate an objective way to estimate sleep activities that traditionally could only be measured with subjective self-report surveys or questionnaires from students or their parents. Therefore, using electronic devices, especially wearable wrist watches or trackers (e.g., Fitbit and the one used here), can be considered an alternative for researchers to investigate relationships between academic performance and sleep activity.

For sleep quality, previous studies have revealed that sleep quality estimated by self-reported Pittsburgh Sleep Quality Index [64] is associated with concurrent GPA [42,47,48,49,51]. Here, we found that the consistency of self-reported sleep quality was positively correlated with the future GPA, but not correlated with the current GPA. In other words, students with consistent sleep quality get a higher future GPA. The absence of the relationship between it and the current GPA suggests that it could not serve as an indicator of short-term academic performance.

These findings observed here collectively demonstrate the importance of using a longitudinal design to examine whether sleep activity predicts future academic performance. Early work has already tried to examine this issue [59,60,61,62,63]. Three studies reported significant associations between sleep and long-term academic performance [59,60,63], whereas two studies did not [61,62]. These studies employed self-report or parent/teacher-report questionnaires to estimate sleep activity and academic performance [63]. Here, we endorse objective sleep measures estimated by wearable devices.

Limitations of the current study need to be pointed out. First, the sample size (n = 45) was relatively small. Using wearable devices similar to those used in the current study, Okano et al. [43] analyzed sleep data from 88 college students. Results from studies with a sample size much larger than the current study are needed to verify the findings observed here. Second, in order not to overwhelm the participants by having them complete daily lengthy questionnaires, and also to ensure the participants could continue to provide reliable feedback, we employed a simplified in-house questionnaire with only a few items to probe into the different aspects of sleep activity. Previous studies have used the Pittsburgh Sleep Quality Index [64] to estimate sleep quality and duration, and the Epworth Sleepiness Scale [86] to estimate daytime sleepiness. Each of the two has multiple items, and provides different sub-measures, and they have been widely used in sleep studies. The in-house questionnaire used here may not be representative, and could not capture the multi-facets of sleep. Future studies with comprehensive questionnaires are needed. Third, other measures, such as students’ mental health (e.g., subclinical depression and anxiety), were not collected in and after the sleep tracking period. The relationships between sleep and academic performance may have been influenced by these factors [79,80,87,88]. Personality traits (e.g., Type D) and physical and sleep characteristics (e.g., Body Mass Index, snoring, and obstructive sleep apnea) also contribute to sleep quality and disturbances [89,90,91]. Complex relationships among them can be further explored in future studies. Finally, the current longitudinal study did not collect data on students’ sleep activity measured by wearable devices and self-report questionnaires across the entire college education. It will be important to examine whether nighttime sleep awakening frequency, self-reported daytime wakefulness, and sleep quality persist after the sleep tracking period, and whether their consistency predicts academic performance [63].

## 5. Conclusions

Using wearable smartwatches and a self-report questionnaire, we provide evidence that nighttime sleep awakening frequency and its consistency can predict long-term academic performance, and self-reported daytime wakefulness and sleep quality can serve as indicators of academic performance. This study demonstrates the usefulness of employing a longitudinal design to examine how sleep activity predicts future academic performance.

## Figures and Tables

**Table 1:** Basic statistical results of average and consistency of sleep measures provided by the wearable device and self-reported measures.

	Average across Tracking Period				Consistency across Tracking Period			
Measures	Min	Max	Mean	SD	Min	Max	Mean	SD
Heart rate	43.54	76.92	57.02	6.66	1.41	12.09	4.41	2.15
Heart rate minimum	36.46	62.00	52.05	5.25	.83	23.48	5.00	4.67
Heart rate maximum	51.23	144.62	122.98	14.31	4.70	30.57	15.90	6.21
Sleep score	72.53	119.92	80.36	6.78	1.00	13.02	5.77	2.35
Sleep time (mins)	305.00	491.46	404.36	45.23	21.82	194.82	87.07	34.83
Deep sleep time (mins)	97.29	249.77	135.21	27.03	16.74	77.36	39.68	14.99
Light sleep time (mins)	128.62	247.71	183.62	29.51	20.97	128.41	49.85	20.96
Rapid eye movement time (mins)	54.94	116.31	83.17	13.79	16.93	74.80	30.46	11.15
Awakening frequency	0	2.29	0.81	0.49	0	2.14	0.85	0.37
Self-reported sleep quality	2.85	8.69	5.83	1.22	0.80	3.69	1.93	0.70
Self-reported daytime tiredness	1.69	8.12	5.19	1.28	0.70	3.64	2.02	0.61
Self-reported daytime wakefulness	0	1.00	0.66	0.30	0	0.96	0.40	0.26

**Table 2:** Pearson’s correlations among measures for averaged data (below the diagonal line) and measures for consistency (above the diagonal line).

	1	2	3	4	5	6	7	8	9	10	11	12	13	14
1		0.758 ***	0.012	−0.041	0.096	−0.038	−0.219	−0.268 ^Ψ^	−0.022	−0.053	−0.133	−0.237	−0.258 ^Ψ^	−0.188
2	0.758 ***		−0.076	−0.110	0.081	−0.031	−0.187	−0.143	0.000	−0.052	−0.312 *	−0.323 *	−0.280 ^Ψ^	−0.089
3	−0.044	−0.141		0.339 *	0.168	0.055	−0.126	−0.160	−0.071	−0.072	−0.210	−0.123	−0.071	0.009
4	0.081	0.040	0.473 ***		0.045	0.203	−0.162	−0.132	−0.228	−0.205	−0.113	−0.063	0.001	0.194
5	−0.212	−0.162	−0.364 *	0.181		−0.219	−0.032	0.021	−0.052	0.000	−0.203	0.050	0.098	0.206
6	−0.040	−0.061	0.389**	−0.417 **	−0.692 ***		0.305 *	0.328 *	0.070	0.308 *	0.245	−0.062	−0.170	−0.101
7	−0.107	0.110	−0.360 *	−0.176	0.123	0.094		0.642 ***	0.748 ***	0.436 **	0.260 ^Ψ^	0.406 **	0.318 *	0.118
8	−0.287 ^Ψ^	−0.125	−0.243	−0.328 *	0.145	0.143	0.572 ***		0.393 **	0.658 ***	0.153	0.305*	0.148	0.156
9	0.119	0.267 ^Ψ^	−0.227	0.078	0.000	−0.003	0.715 ***	−0.094		0.395 **	0.212	0.203	0.163	−0.113
10	−0.006	0.053	−0.097	−0.080	0.107	0.085	0.595 ***	0.186	0.348 *		0.270 ^Ψ^	0.100	−0.030	−0.125
11	−0.165	−0.387 **	0.066	−0.044	0.131	0.095	0.075	−0.056	0.134	0.086		0.265 ^Ψ^	0.228	0.017
12	0.122	−0.030	−0.132	−0.102	−0.014	0.018	0.277 ^Ψ^	0.148	0.298 *	0.181	−0.068		0.792 ***	0.629 ***
13	−0.053	−0.189	−0.182	−0.225	0.004	0.096	0.167	0.095	0.210	−0.118	0.121	0.611 ***		0.548 ***
14	0.309 *	0.359 *	−0.103	0.094	−0.243	0.078	0.356 *	0.057	0.459 **	0.073	−0.331 *	0.407 **	0.123	

Note: 1, current GPA; 2, future GPA; 3, heart rate; 4, heart rate minimum; 5, heart rate maximum; 6, sleep score; 7, sleep time (mins); 8, deep sleep time (mins); 9, light sleep time (mins); 10, rapid eye movement time (mins); 11, awakening frequency; 12, self-reported sleep quality; 13, self-reported daytime tiredness; 14, self-reported daytime wakefulness; ^Ψ^ *p* < 0.10, * *p* < 0.05, ** *p* < 0.01, *** *p* < 0.001.

**Table 3:** Hierarchical regression analyses predicting cumulative GPA from semester GPA and nighttime sleep awakening frequency.

		Future GPA	
Step	Variables	R^2^	ΔR^2^
1	Current GPA	0.575	0.575 ***
2	Nighttime sleep awakening frequency	0.646	0.071 **
2	Consistency of nighttime sleep awakening frequency	0.620	0.045 *

*Note.* * *p* < 0.05. ** *p* < 0.01. *** *p* < 0.001.

## Data Availability

Data are available upon request.
